# Automatic Inside Point Localization with Deep Reinforcement Learning for Interactive Object Segmentation

**DOI:** 10.3390/s21186100

**Published:** 2021-09-11

**Authors:** Guoqing Li, Guoping Zhang, Chanchan Qin

**Affiliations:** 1 College of Physical Science and Technology, Central China Normal University, NO. 152 Luoyu Road, Wuhan 430079, China; liguoqing@mails.ccnu.edu.cn (G.L.); gpzhang@mail.ccnu.edu.cn (G.Z.); 2 Key Laboratory of Quark and Lepton Physics (MOE) and College of Physics Science and Technology, Central China Normal University, NO. 152 Luoyu Road, Wuhan 430079, China; 3 School of Big Data and Computer Science, Guizhou Normal University, The University Town, Guian New Area, Guiyang 550025, China; 4 Center for RFID and WSN Engineering, Department of Education, Guizhou Normal University, The University Town, Guian New Area, Guiyang 550025, China

**Keywords:** interactive image segmentation, Markov Decision Process (MDP), Deep Reinforcement Learning (DRL), inside point localization, Deep Q-Network (DQN)

## Abstract

In the task of interactive image segmentation, the Inside-Outside Guidance (IOG) algorithm has demonstrated superior segmentation performance leveraging Inside-Outside Guidance information. Nevertheless, we observe that the inconsistent input between training and testing when selecting the inside point will result in significant performance degradation. In this paper, a deep reinforcement learning framework, named Inside Point Localization Network (IPL-Net), is proposed to infer the suitable position for the inside point to help the IOG algorithm. Concretely, when a user first clicks two outside points at the symmetrical corner locations of the target object, our proposed system automatically generates the sequence of movement to localize the inside point. We then perform the IOG interactive segmentation method for precisely segmenting the target object of interest. The inside point localization problem is difficult to define as a supervised learning framework because it is expensive to collect image and their corresponding inside points. Therefore, we formulate this problem as Markov Decision Process (MDP) and then optimize it with Dueling Double Deep Q-Network (D3QN). We train our network on the PASCAL dataset and demonstrate that the network achieves excellent performance.

## 1. Introduction

Interactive image segmentation allows users to explicitly control the segmentation mask using human-friendly annotators, which can be formalized via various representations: bounding boxes, scribbles, clicks, or extreme points. As one of the fundamental problems in computer vision, it has obtained remarkable results in broad applications, such as medical image analysis [1], image editing [2], and especially pixel-level annotation [3]. In the early days, a large number of traditional approaches [4,5,6,7,8] have been developed in this direction. Boykov et al. [4] considered interactive segmentation problem as an optimization problem and utilized a graph cut-based method to extract the object automatically. Following, Price et al. [6] improve the graph cut method by applying geodesic distances for energy minimization. Grady introduces an interactive segmentation algorithm called random walks [7]. Here, the pixel labels are assigned as the label of the first seed that the walker reaches. All these methods based on low level-features cannot distinguish between the target object and background in the case of complex and variable scenes.

Over the past few years, deep learning-based algorithms have become popular in computer vision and have also showed astonishing performances in interactive segmentation problems. Xu et al. [9] put forward a CNN-based model to solve the interactive segmentation problem, which is one of the pioneering works in this field. Liew et al. [10] utilized local information surrounding the user annotations to get a precise segmentation mask. Li et al. [11] generated multiple hypothesis segmentations and thus refined the network predictions. To further improve the performance, Mahadevan et al. [12] introduced an effective iterative training algorithm for interactive image segmentation. BRS [13] and f-BRS [14] both applied a backpropagating refinement framework for optimization in this area. Maninis et al. [15] developed a novel interactive manner that required user annotations on a tight target object.

**Figure 1:**
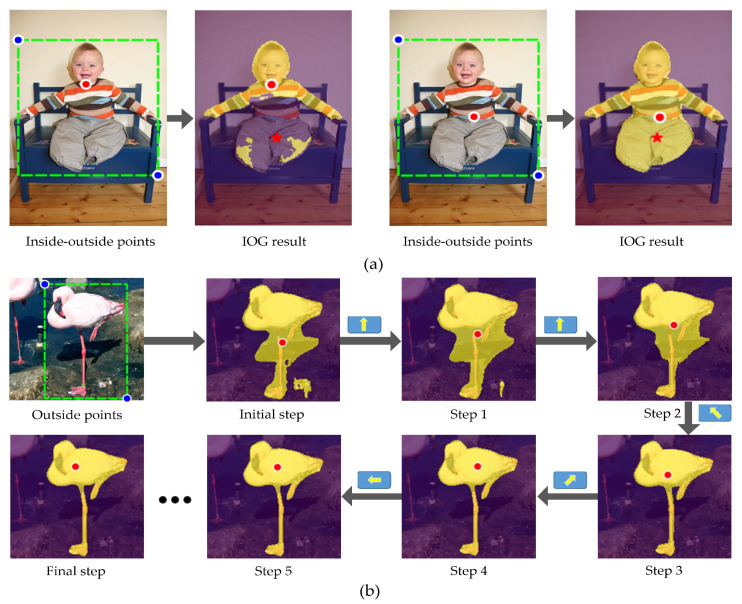
The blue and red dots represent the outside and inside points, respectively. (**a**) IOG [16] segmentation example with different inside points. “★” denotes the simulated inside point located at the object center. (**b**) Automatic inside point localization process through the IPL-Net. At each step, the IPL-Net moves the inside point to a new location.

Recently, Zhang et al. [16] explored the use of the inside and outside points for interactive segmentation. Despite its simplicity, the Inside-Outside Guidance (IOG) algorithm has shown fast clicking speed and excellent segmentation results across different application domains. However, we observe that the inconsistent choices in selecting the inside point between training and testing will often result in significant performance degradation. From Figure 1a, we can observe a large performance degradation when there is significant inconsistent selection between the annotations of the inside point and the simulated inside point. Therefore, it is meaningful to enable the machine to locate the inside point automatically.

To tackle the aforementioned issues as well as to reduce human effort, we propose an approach named IPL-Net, which stands for Inside Point Location Network. When a human annotates two outside points that form a rectangle encircling the target object of interest, the ultimate goal of the proposed system is to infer the suitable position for the inside point to help the IOG algorithm for accuracy segmentation results, as illustrated in Figure 1b. Here, the clicked exterior points can be considered as an incomplete annotation for the IOG algorithm, where the inside point is missing; our proposed system automatically generates the sequence of movement to localize the inside point. In this study, the problem is difficult to define as a supervised learning framework, because it is expensive to collect images and their corresponding inside points. Hence, we formulate the inside point localization problem as Markov Decision Process (MDP) and then propose a reinforcement learning framework for teaching computers to accomplish this task. Our approach is inspired by [17], which also proposed a Deep Reinforcement Learning framework for interactive segmentation task. However, our proposed method differs from theirs in the following aspect. Song et al. used the deep reinforcement agent for generating artificial seed points, while we apply a sequence of movements to the inside point that initially located at the geometric center of the cropped image and finally at a suitable position. The sequence of movements is decided by an agent that analyzes the content of the current observation to select the next best action. More concretely, our agent starts by analyzing the cropped image, the initial position of the inside point, and the segmentation results produced with the initial Inside-Outside Guidance information, and then determines the direction of the inside point’s movement. After the inside point moves to a new position, our agent uses the new inside guidance information and segmentation results as a next input and repeats the process of moving the inside point.

To imitate the intention of the inside point localization agent, we develop a novel reward function considering the Intersection-over-Union (IoU) score of the segmentation results and the Centrality Degree (CD) of the inside point. The agent was trained using Dueling Double Deep Q Network (D3QN) [18,19,20], which copes well with the discrete action spaces. We conduct comprehensive experiments on PASCAL VOC [21], GrabCut [5] and COCO [22] datasets. Results and analyses of comparative prove the effectiveness of our propose IPL-Net. Comparing with interactive IOG segmentation, we further reduce the number of clicks required from three to two.

## 2. Related Work

Interactive IOG Segmentation: In this section, we briefly introduce the Interactive Inside-Outside Guidance (IOG) segmentation method. In an IOG algorithm, the workload of the annotator is reduced to only providing three points: an inside point located around the center of the object and two outside points at any symmetrical corner locations that form an almost-tight bounding box enclosing the target object of interest. Then, two separate heatmaps for the foreground and background annotations are created by encoding as Gaussians map. In doing so, the inside and outside heatmaps are concatenated as two extra channels to the cropped RGB image, which then serves as an input to the segmentation network. Moreover, Zhang et al. proposed a coarse-to-fine framework for obtaining more precise segmentation masks. Despite its simplicity, the IOG algorithm has achieved high performance across different application domains. However, we observe that it is difficult for users to reach a consensus between training and testing when annotating the inside point, resulting in significant performance degradation. To deal with this problem, we propose to find the suitable position for the inside point automatically and train the network using Deep Reinforcement Learning. This is the idea behind the Inside Point Location Network (IPL-Net).

Deep Reinforcement Learning: In recent years, Deep Learning techniques have shown their powers in extendibility and performance of machine learning. One typical application is the sequential decision-making task of Reinforcement Learning (RL), where an agent learns an optimal policy from interacting with the environment by taking actions and receiving observations and rewards. Researchers in DeepMind technologies proposed Deep Q Network (DQN) [18] and kicked the door of Deep Reinforcement Learning (DRL) via combining Q-learning with a deep neural network. Techniques such as Double DQN (DDQN) [19] and Dueling DQN [20] have been developed to improve the performance of the DQN algorithm. The former was introduced to solve the over-estimation issue and yield accurate value estimates to improve the performance of the model, while the latter was used for the purpose of learning the state-value function efficiently, where the representation of state values and action advantages are separated. Following, Deep Deterministic Policy Gradient (DDPG) [23] has shown better results to resolve the difficulties caused by high-dimensional continuous action space. It combines the actor network with critic network. Another notable Actor-Critic algorithm is A3C [24], which has multiple agents executing in parallel. Soft Actor-Critic (SAC) algorithm [25] attempted to maximize expected reward while also maximizing entropy. This provided sample-efficient learning while retaining the benefits of stability. TD3 [26] developed a novel variant of Double Q-learning algorithm to solve the overestimation problem in actor-critic methods. In recent years, DRL has also found applications in the field of computer vision [27], robotics [28], wireless transmission [29], and other complex problems [30].

In this paper, we investigate the application of the Deep Reinforcement Learning-based method to the interactive image segmentation framework. We adopt Dueling Double Deep Q Network (D3QN) since there are a total of nine choices of actions. In addition, we also use the concept of Prioritized Experience Replay [31], which replaced DQN’s uniform sampling strategy from the replay memory with a novel sampling strategy to accelerate learning dramatically. Following, the description of the proposed automatic inside point location system is provided in Section 3. Further, results and ablation experiments are shown in Section 4. Section 5 and Section 6 include conclusions for our IPL-Net framework along with a discussion on future perspectives.

## 3. Automatic Inside Point Localization System

### 3.1. Overview

In this paper, we present a novel automatic inside point localization system to help the interactive IOG segmentation, which was used as an off-the-shelf segmentation algorithm in our proposed system. We call it IPL-Net. Different from IOG algorithm, our IPL-Net requires only two exterior points clicked at any symmetrical corner locations to indicate the object of interest. Following, we consider these two exterior points as an incomplete annotation for the IOG algorithm, where the inside point is missing. Therefore, the ultimate goal of our proposed IPL-Net is to infer the suitable position of the inside point for the IOG algorithm. We formulate this problem as a Markov Decision Process (MDP) and then optimize it by reinforcement learning paradigm, more specifically Dueling Double Deep Q Network (D3QN), which copes well with the discrete action space of the agent.

**Figure 2:**
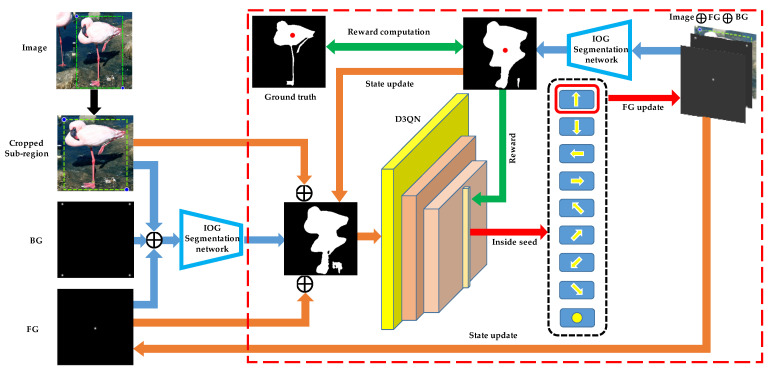
Overview of the proposed IPL-Net. The cropped image, the inside guidance information, and the binary masks are the input of the D3QN. The inside point is updated using the action from the D3QN, and the segmentation mask is obtained using the new inside-outside guidance information. The new segmentation result and the position of the inside point are used to compute the reward, and this process is repeated for a fixed number of steps. The orange, red, and green arrows represent behaviors related to state, action, and reward, respectively. The black rectangle represents the action range.

The overview of our algorithm is shown in Figure 2. We initially set the position of the inside point at the geometry center of the cropped image. The operation of our framework deals with the cropped image and the initial inside-outside guidance information. By utilizing the five-channel input information, performing the interactive IOG method yields a segmentation result. The segmentation result is concatenated with the cropped RGB image, the Gaussian map of the current inside point, to also form a five-channel input for the D3QN framework. At each step, the inside point must decide which direction to move. When it moves to the next location, the Gaussian map of the inside point is updated. After creating a new binary mask by the new inside-outside guidance information, our agent uses this predict result and the new inside guidance information as a next input and repeats the sequence of cyclic operations for a fixed number of steps. The segmentation results and the position of the inside point are used for two purposes: First, the acquired binary masks and the inside guidance information are used as a state of the next iteration. The second is to compute the reward signal that used to update the network.

### 3.2. The Model

The core part of the proposed IPL-Net is to find the suitable position for the inside point by the agent to help the interactive IOG algorithm. We cast this problem as a Markov Decision Process (MDP) consisting of state, action, and reward. This section describes details of these components.

State: It is necessary to design an effective state representation to help the agent to make the best choice. For our problem, the cropped RGB image contains important features. Additionally, the information that can be taken from the observation changes at each step. At each step, two kinds of information can be achieved when the inside point moves to a new position: one is the Gaussian map of the inside point, and the other is a binary segmentation mask using the interactive IOG segmentation algorithm. As a result, the state is defined as the cropped RGB image, the Gaussian map of the inside point, and the segmentation mask. Formally, $S_{t}=(I_{c},g_{t},m_{t})$, $g_{t}$ and $m_{t}$ are binary images. Unlike many existing algorithms, our proposed framework does not use any past observations and only makes use of the current observation as the state.

Action: In this study, our goal is to find the suitable position for the inside point. If we define the action corresponding to all the pixels in the cropped image, the action space becomes too large, leading to training problems. Therefore, our algorithm simplifies this method by defining the action as the direction of the inside point’s movement given the states, and we set the origin position of the inside point at the geometry center of the cropped image. At each step, the agent moves the inside point in the horizontal and vertical axes or remains stationary. This way, there are a total of nine kinds of choices, including go up, down, left, right, left-up, left-down, right-up, right-down, and still. The position of the inside point is represented by the coordinates of pixel: $p=[x_{in},y_{in}]$. Any of the actions make a change to the position of the inside point in the following way:

(1) $$m_{x}=\frac{c_{w}}{a}\;m_{y}=\frac{c_{h}}{a},$$

where $c_{w}$ and $c_{h}$ denote the width and height of the cropped image. At each step, the agent makes a decision to move the inside point to a new position or remains stationary. If an agent selects one of the first eight actions, the inside point moves to the corresponding location by adding or subtracting $m_{x}$ or $m_{y}$ to the x or y coordinates. For instance, the action right-up adds $m_{x}$ to $x_{in}$, while subtracting $m_{y}$ to $y_{in}$. We set a=15 in all our experiments. We terminate the process when the agent takes the still action or after moving 10 steps without using the still action. We believe that for most objects, 10 steps is enough to localize the position of the inside point that is initially located at the geometry center of the cropped image.

Reward: The design of reward has a significant impact for training the agent. In our system, the results of agent action are the new position of the inside point and segmentation mask. Thus, improvement in our setup can be determined by a comparison of the segmentation mask with the Ground Truth (GT). As a common metric, Intersection-over-Union (*IoU*) between the binary masks and the Ground Truth is measured as:

(2) $$IoU=\frac{area(b)\cap area(g)}{area(b)\cup area(g)},$$

where b means the binary segmentation result and g means the ground truth mask. We define the *IoU* reward using the change trend of *IoU*:

(3) $$r_{IoU}=IoU(b_{t},g)-IoU(b_{t-1},g),$$

where $b_{t}$ and $b_{t-1}$ are the segmentation result in the $t^{th}$ and $t-1^{th}$ step.

According to IOG algorithm, the position of the object center is simulated as the inside point during training. To encourage the agent to move to the center of the target object, we additionally consider the Centrality Degree (CD) [3] of the inside point to assist the *IoU* reward. We define ε(p) to measure the distance between the inside point p and the foreground center, which can be expressed as follows:

(4) $$\varepsilon (p)=\frac{\phi (p,R_{n})}{\max_{\forall p_{0}\in R_{p}}\phi (p_{0},R_{n})},$$

where ${\mathbb{R}}_{p}$ and ${\mathbb{R}}_{n}$ denote the sets of pixels in foreground and background according to the Ground Truth, p being the inside point. $\phi (p,R_{n})$ represents the shortest distance from the inside point p to the background region, which is formulated as:

(5) $$\phi (p,{\mathbb{R}}_{n})=\underset{\forall p_{s}\in {\mathbb{R}}_{n}}{\min}d(p,p_{s}),$$

where $d(p,p_{s})$ means the Euclidean distance between point p and $p_{s}$. The IOG algorithm chooses the point whose ε(p)=1 in cropped images as the simulated inside point. The CD reward is also estimated using the differential of CD from one state to another, formulated as:

(6) $$r_{CD}=\varepsilon_{t}(p)-\varepsilon_{t-1}(p).$$

In this work, the reward function at the t-th step is defined as a weighted sum of two factors:

(7) $$r_{our}=\alpha r_{iou}+\beta r_{CD}$$

where $r_{iou}$ and $r_{CD}$ are the corresponding reward, and α and β are used to adjust the range of reward. We empirically set α and β to 0.7 and 0.3. In this way, the agent pays a penalty for decreasing *IoU* and moving the inside point away from the target center.

The still action has a different reward function since it leads to a terminal state that does not change the position of the inside point, and thus, $r_{IoU}$ and $r_{CD}$ will always be zero. So the reward for the still action is defined as follows:

(8) $$r_{s}=\{\begin{array}{c} +2\\ -2 \end{array}\begin{array}{c} if\;IoU(b,g)\geq \eta \\ otherwise \end{array},$$

where s is the still action, and η means the *IoU* obtained by IOG algorithm using the simulated inside point.

### 3.3. Training the Inside Point Localization Agent with Deep Reinforcement Learning

The purpose of the agent is to move the inside point by choosing actions in a way that maximizes the future rewards. In this study, we followed the improved Double DQN algorithm of Hasselt et al. [19] to train the agent. Double DQN uses the same techniques for effective learning as proposed in DQN [18]. First, target network and experience replay techniques were used to improve the stability of the learning; second, the exploration-exploitation was adopted to improve the performance of the algorithm. The only difference was that Double DQN decoupled the selection from the evaluation to solve the overestimate problem that commonly occurs in DQN. This means that we selected the action according to the parameters of the online evaluate network, while we used the offline target network to evaluate the value of the policy.

More specifically, in DQN, the parameters update can be defined with the given state $s_{t}$, action $a_{t}$ and resulting state $s_{t+1}$:

(9) $$\theta_{t+1}=\theta_{t}+\alpha (y_{t}^{Q}-Q(s_{t},a_{t};\theta_{t}))\nabla_{\theta_{t}}Q(s_{t},a_{t};\theta_{t}),$$

where the target $Y_{t}^{Q}$ is formulated as:

(10) $$y_{t}^{DQN}=r_{t+1}+\gamma \underset{a}{\max}Q(s_{t+1},a;\theta_{t}^{-}),$$

here, $r_{t+1}$ denotes the immediate reward when taking action $a_{t}$ at state $s_{t}$. However, Double DQN uses the following target:

(11) $$y_{t}^{DoubleDQN}=r_{t+1}+\gamma Q(s_{t+1},\underset{a}{\arg\max}Q(s_{t+1},a;\theta_{t});\theta_{t}^{-}),$$

where $\theta_{t}$ is the weights for the evaluated network, and $\theta_{t}^{-}$ is the weights for the target network.

In order to improve the performance of the Double DQN algorithm, we adopt the framework of the Dueling DQN [20], where the representation of state values and action advantages are separated. During training, we also apply the trick of Prioritized Experience Replay (PER) [31] to boost the sample efficiency and improve the performance for the agent.

### 3.4. Network Architecture

**Figure 3:**
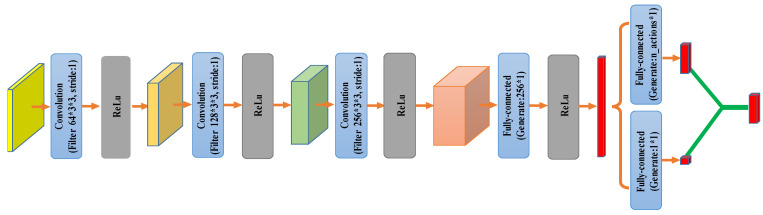
D3QN architecture. The input representations are first down-sampled to 64×64 size. It is processed by the D3QN, which predicts the value of the nine actions.

The cropped RGB image, the inside guidance information, and the binary mask from the IOG segmentation were concatenated to form a five-channel input for the network. Before being fed into deep neural networks, the input representations were first down-sampled to 64×64 size. The neural network consists of a stack of three convolutional layers. The size of the filters are all 3×3 with stride 1, and the number of the filters are 64, 128, and 256, respectively. This is followed by a fully-connected layer and consists of 256 units. All these hidden layers were separated by a rectifier nonlinearity. By using the Dueling network architectures [20], two streams of fully connected layers were performed. The streams were used to predict two separate estimators to represent the state value and the action advantage. Then, performing a fully-connected operation, the advantage function was produced as a nine-dimension output corresponding to the action space size. Simultaneously, the state value function is a constant. Finally, the advantage function is combined with the state value function to obtain a set of Q values, one for each action. The action is determined according to the Q-function having the maximum value. The D3QN architecture is shown in Figure 3.

## 4. Experiments

We now show the practical performance of our proposed IPL-Net on three popular datasets, including PASCAL VOC [21], GrabCut [5], and COCO [22]. We demonstrate that the agent has excellent performance in finding the most suitable position of the inside point for different types of images.

### 4.1. Datasets

PASCAL VOC: We train our proposed IPL-Net on PASCAL 2012. As done in [16], we indicate that the PASCAL train set is augmented with additional images from SBD [32] and the one without SBD images as PASCAL-10k (10,582 images with 25,832 objects) and PASCAL-1k (1464 images with 3507 objects), respectively. For evaluation, we use the validation set in this dataset, which consists of 1449 images with 3427 objects.

GrabCut: GrabCut is a benchmark dataset for interaction segmentation. It contains 50 natural scene images with corresponding masks.

COCO: COCO is a benchmark dataset for object segmentation. This dataset consists of objects in 80 categories.

### 4.2. Network Training

**Figure 4:**
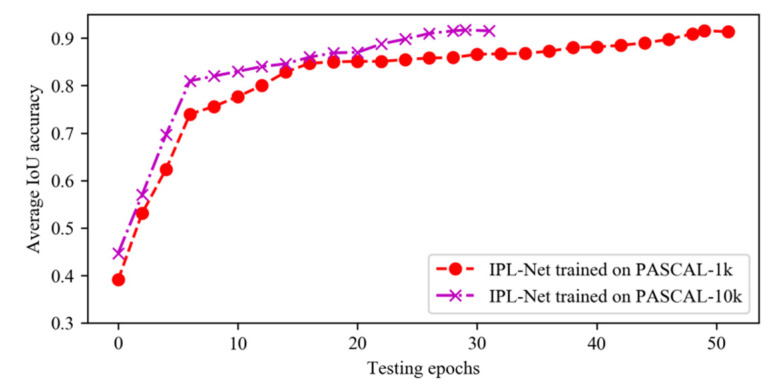
The average IoU accuracy on PASCAL val set.

**Figure 5:**
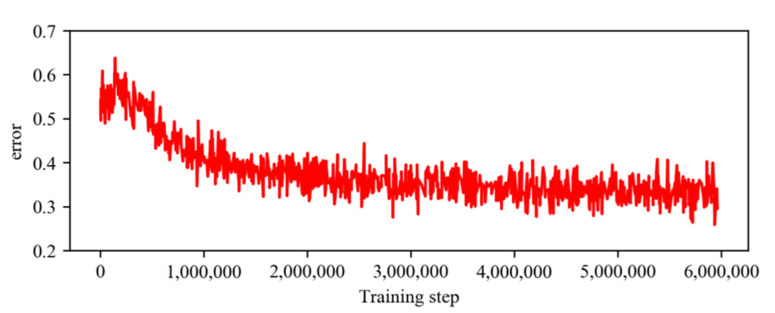
The training error curve trained on PASCAL-10k datasets.

In this study, IPL-Net is trained for PASCAL dataset from scratch. We resize all information to the resolution of 64×64 pixels before feeding into the D3QN network. Before updating the parameters, we store the experience in the buffer during the first 100,000 steps, and we use a replay memory of 400,000 experience for training the agent. Figure 4 shows the performance on PASCAL VOC val set trained on different PASCAL datasets. From this Figure, we can see that the average IoU accuracy on PASCAL val set reaches the maximum at the 50th epoch using the model trained on PASCAL-1k and 30th epoch using the model trained on PASCAL-10k. So we train on PASCAL-1k for a total of 50 epochs or on PASCAL-10k for 30 epochs. We set the minibatch size as 512 and use the Adam [9] algorithm for optimization. For data augmentation methods, we use random horizontal flips, resize images (from 0.75 to 1.25), and rotate images (from −20 to 20). In order to balance exploration and exploitation, we use a ε-greedy policy, which selects a random action with probability ε or an action according to the learned network with probability 1−ε. During training, the probability ε for the exploration of random action is annealed from 0.9 to 0.1 over the first 100,000 steps, and fixed at 0.1 thereafter. It takes about 0.9 s to find the most suitable location of the inside point on a single NVIDIA 2080Ti GPU and 3.60 GHz Intel Core i9-9900K CPU (Xi’an Kunlong Computer Technology Co. LTD, Xi’an, China). All our experiments are conducted on the public PyTorch platform. In all experiments, we use the pretrained segmentation model provided by the authors [16]. We denote the model trained on PASCAL-1k and PASCAL-10k as PASCAL and PASCAL_SBD, respectively. We show the training error curve of the agent trained on PASCAL-10k datasets in Figure 5. For better visualization, each point represents the average error value for 100 steps.

### 4.3. Interactive Segmentation Results

**Figure 6:**
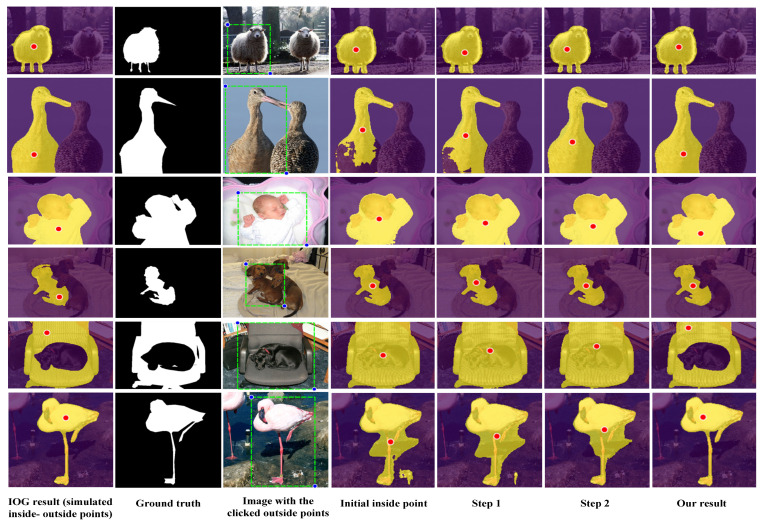
The results on PASCAL val set. The first three columns show the simulated inside-outside points with corresponding IOG result, the ground truth mask, and the image with the clicked outside points (blue dots). The right four columns show the initial inside point, the inside point of the first two steps, and the inside point of the final step with corresponding IOG result, respectively. Each segmentation result and the corresponding inside point are overlaid on the input image.

First, we evaluate the performance on PASCAL VOC val set to validate the effectiveness of our IPL-Net. We set the geometric center of the cropped image as the initial position of the inside point. Figure 6 shows sequences of segmentation results and the inside point in first two steps and the final step. As shown in Figure 6, the results show that the segmentation performance is significantly improved when the inside point moves according to the proposed IPL-Net compared with the IOG segmentation corresponding to the initial inside point. Notice that the actions chosen attempt to move the inside point to close to the simulated one, and also to help the interactive IOG segmentation algorithm to obtain accuracy in the segmentation result. The agent also has the ability to find the optimal position of the inside point when there are unrelated objects lying inside the target object, as in the case of the five rows.

Comparison with outside points only based methods: We compare our IPL-Net with the following methods: outside only [16] and the two-stage [16]. The outside only algorithm trains a network that implicitly takes the geometry center of the tight box formed by the outside points as the inside point and passes to IOG for segmentation. Then the two-stage algorithm uses a simple network to predict coarse segmentation results and then extracts the inside point from the segmentation results to obtain the final binary masks. Refer to [16] for more details. We directly cite the IoU accuracy reported in the papers. Table 1 shows the segmentation results on PASCAL val using outside points annotations only. It can be observed that our IPL-Net outperforms the other two algorithm by more than 0.8% and 0.6%, which well validate the effectiveness of our proposed method in inferring the position of the inside point to help the IOG algorithm to achieve accurate performance.

### 4.4. Comparison with the State of the Arts

We compare our proposed algorithm with several state-of-the-art algorithms over two datasets. Table 2 shows the mean number of clicks that each algorithm requires to achieve a certain IoU accuracy on PASCAL and GrabCut datasets. We set the target IoU score to 85% or 90% for different datasets, denoting the corresponding measures as PASCAL@85 and GrabCut@90 respectively. As show in this table, we can see that our proposed IPL-Net requires only two clicks to reach the specific accuracy on both datasets. The competitive results well demonstrate the effectiveness of our IPL-Net.

Next, we validate the generalization ability of our proposed IPL-Net on GrabCut and COCO mini-val set. We leverage the D3QN model trained on PASCAL-10k and infer the IoU on COCO MVal (regardless of the categories), COCO MVal seen (the same categories as training), COCO MVal unseen (different categories as training), and GrabCut dataset. Table 3 shows the results compared with the IOG algorithm (simulated inside point). It can be observed that our IPL-Net achieves a little performance degradation when using only two clicks. It is to be noticed that the result of the IOG is an ideal value. As discussed in [16], the inconsistent inputs between training and testing when annotating the inside point will often have a negative effect on the segmentation performance. Therefore, it is meaningful that our IPL-Net can locate the inside point automatically.

### 4.5. Ablation Experiments

Pretrained model and training dataset: We perform ablation experiments on PASCAL VOC val set to analyze how the components affect the performance of the agent. Specifically, we study the performance of our proposed IPL-Net when using different pretrained segmentation models and a different number of training images. We conduct experiments using two pretrained models provided by the authors, i.e., PASCAL and PASCAL_SBD. As shown in Table 4, the pretrained model ‘PASCAL_SBD’ can respectively lead to a performance improvement of 1% under the PASCAL-1k training dataset. When using the PASCAL-10k to train the D3QN network, the performance can be further improved from 91.5% to 91.7%.

**Figure 7:**
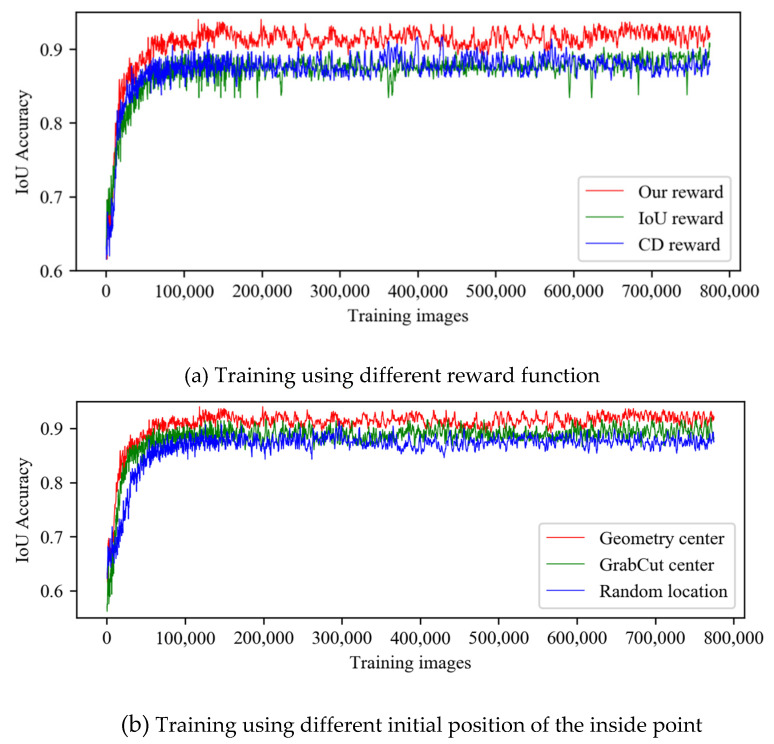
Ablation experiments. (**a**) shows IPL-Net learning progress using different reward function. (**b**) shows IPL-Net learning progress using different initial positions of the inside point.

Reward: To verify the effectiveness of the proposed reward function, we consider our D3QN trained with the reward $r_{IoU}$, $r_{CD}$, and $r_{our}$ described in Section 3.2 respectively. The change in IoU accuracy of the training set according to the learning steps is shown in Figure 7a. For better visualization, each point represents the average IoU value for 100 images. As shown in Figure 7a, we can observe that our proposed reward function is a better metric in finding the suitable position of the inside point.

Initial position of the inside point: In this section, we analyze the differences between the results obtained by our algorithm when we use different initial positions of the inside point. We show the performance of agents trained with different initial position of inside point in Figure 7b. The ‘Geometry center’ is initially located at the inside point at the geometry center of the cropped image; the ‘GrabCut center’ is initially located at the inside point at the center of the segmentation result following GrabCut algorithm [5]; the ‘Random location’ is initially located at the inside point with random selection. Based on the IoU accuracy, the ‘Geometry center’ is significantly better than the other two methods. This is because we set the maximum number of the inside point’s movement to 10. Therefore, the other two initial methods sometimes cannot move to the suitable position in 10 steps if the initial position of the inside point is far away from the simulated position of the inside point.

## 5. Discussion

In this paper, a Deep Reinforcement Learning-based method, named IPL-Net, is explored to infer the suitable position for the inside point to help the IOG algorithm. When users annotate two outside points that form a rectangle encircling the target object of interest, our proposed system automatically generates the sequence of movement to localize the inside point. We then perform the IOG interactive segmentation method for precisely segmenting the target object. The experimental results on three popular datasets indicate that our proposed IPL-Net has excellent performance in finding the most suitable position of the inside point for different types of images. Notice that our goal is not to train the binary mask directly, but to train the inside point localization step that can help the existing interactive IOG segmentation algorithm. Part of our future work includes training the system using more effective DRL algorithm for improving the accuracy of the localization for the inside point.

## 6. Conclusions

A system that learns to localize the inside point for assisting the IOG algorithm to segment an object accurately was presented. The goal was to reduce the workload of the interactive annotation. Due to the high cost of collecting the images and their corresponding inside points, it is difficult to define this problem as a supervised leaning framework. Therefore, we formulate the inside point localization as a Markov Decision Process (MDP) problem. Deep Reinforcement Learning demonstrated to be an effective way to learn a localization policy. The proposed agent learnt from its own mistakes and optimized the policy to find the most suitable position for the inside point.

## Figures and Tables

**Table 1:** Comparison with outside points-based methods.

Method	Outside Only [16]	2-Stage [16]	IPL-Net
IoU	90.9	91.1	91.7

**Table 2:** The mean number of clicks required to achieve the specific IoU accuracy in PASCAL and GrabCut dataset. Lower is better.

Methods	Number of Clicks	
	PASCAL@85%	GrabCut@90%
Graph cut [4]	>20	>20
Geodesic matting [8]	>20	>20
Random walker [7]	16.1	15
iFCN [9]	8.7	7.5
RIS-Net [10]	5.7	6
DEXTR [15]	4	4
IOG [16]	3	3
IPL-Net(ours)	2	2

**Table 3:** The generalization ability of our proposed IPL-Net.

Train	Test	IOG [16]	Ours
PASCAL-10k	COCO Mval	81.9	81.3
PASCAL-10k	COCO Mval(seen)	81.7	81.3
PASCAL-10k	COCO Mval(unseen)	82.1	81.5
PASCAL-10k	GrabCut	96.3	95.9

**Table 4:** Ablation experiments. Justification of the pretrained model and the number of training images on the PASCAL VOC 2012 val set.

Pretrain Model	Train	Test	IOG [16]	Ours
PASCAL	PASCAL-1k	PASCAL	92.0	90.5
PASCAL_SBD	PASCAL-1k	PASCAL	93.2	91.5
PASCAL_SBD	PASCAL-10k	PASCAL	93.2	91.7

## Data Availability

The data presented in this study are publicly available. The PASCAL dataset can be found here: http://host.robots.ox.ac.uk/pascal/VOC/voc2012/ (accessed on 10 September 2021); the MSCOCO dataset can be found here: http://cocodataset.org (accessed on 10 September 2021); the GrabCut dataset can be found here: http://research.microsoft.com/en-us/um/cambridge/projects/visionimagevideoediting/segmentation/grabcut.htm. (accessed on 10 September 2021).
